# (η^6^-1-Isopropyl-4-methyl­benzene)­bis­(η^1^-pyridin-2-ylethynido-κ*C*)(tri­methyl­phosphane-κ*P*)ruthenium(II)

**DOI:** 10.1107/S241431462601045X

**Published:** 2026-10-20

**Authors:** Bre-Anna N. Willis, Aaron Meeks, Colin D. McMillen, Jared A. Pienkos, John P. Lee

**Affiliations:** a The University of Tennessee at Chattanooga Department of Chemistry #2252 615 McCallie Avenue Chattanooga TN 37403 USA; b Clemson University, Department of Chemistry, Clemson, SC 29634, USA; Goethe-Universität Frankfurt, Germany

**Keywords:** crystal structure, ruthenium, 2-ethnyl­pyridine, *para*-cymene

## Abstract

The title complex, [Ru(C_7_H_4_N_2_)_2_(C_10_H_14_)(C_3_H_9_P)] or (*p*-cym)Ru(C_2_-2py)_2_(PMe_3_), where *p*-cym = η^6^-1-isopropyl-4-methyl­benzene and C_2_-2py = η^1^-*C*-pyridin-2-ylethynide, comprises a pseudo-octa­hedral Ru^II^ atom with *p*-cym occupying one face of the octa­hedron and the other face comprised of monodentate PMe_3_ and two η^1^-C_2_-2py ligands. The Ru—C_ethynide_ bond lengths are 2.0248 (16) and 2.0343 (17) Å. The N—C—C—N torsion angle of 151.1 (2)° suggests a near anti-conformation for the two pyridine rings. In the crystal, hydrogen bonding involving one C—H donor and one nitro­gen acceptor from each pyridine ring generates dimers propagating along the *a*-axis direction *via* supporting C—H⋯π inter­actions.

## Abstract 2

The title complex, [Ru(C_7_H_4_N_2_)_2_(C_10_H_14_)(C_3_H_9_P)] or (*p*-cym)Ru(C_2_-2py)_2_(PMe_3_), where *p*-cym = η^6^-1-isopropyl-4-methyl­benzene and C_2_-2py = η^1^-*C*-pyridin-2-ylethynide, comprises a pseudo-octa­hedral Ru^II^ atom with *p*-cym occupying one face of the octa­hedron and the other face comprised of monodentate PMe_3_ and two η^1^-C_2_-2py ligands. In the crystal, hydrogen bonding involving one C—H donor and one nitro­gen acceptor from each pyridine ring generates dimers propagating along the *a*-axis direction *via* supporting C—H⋯π inter­actions.

## Graphical abstract

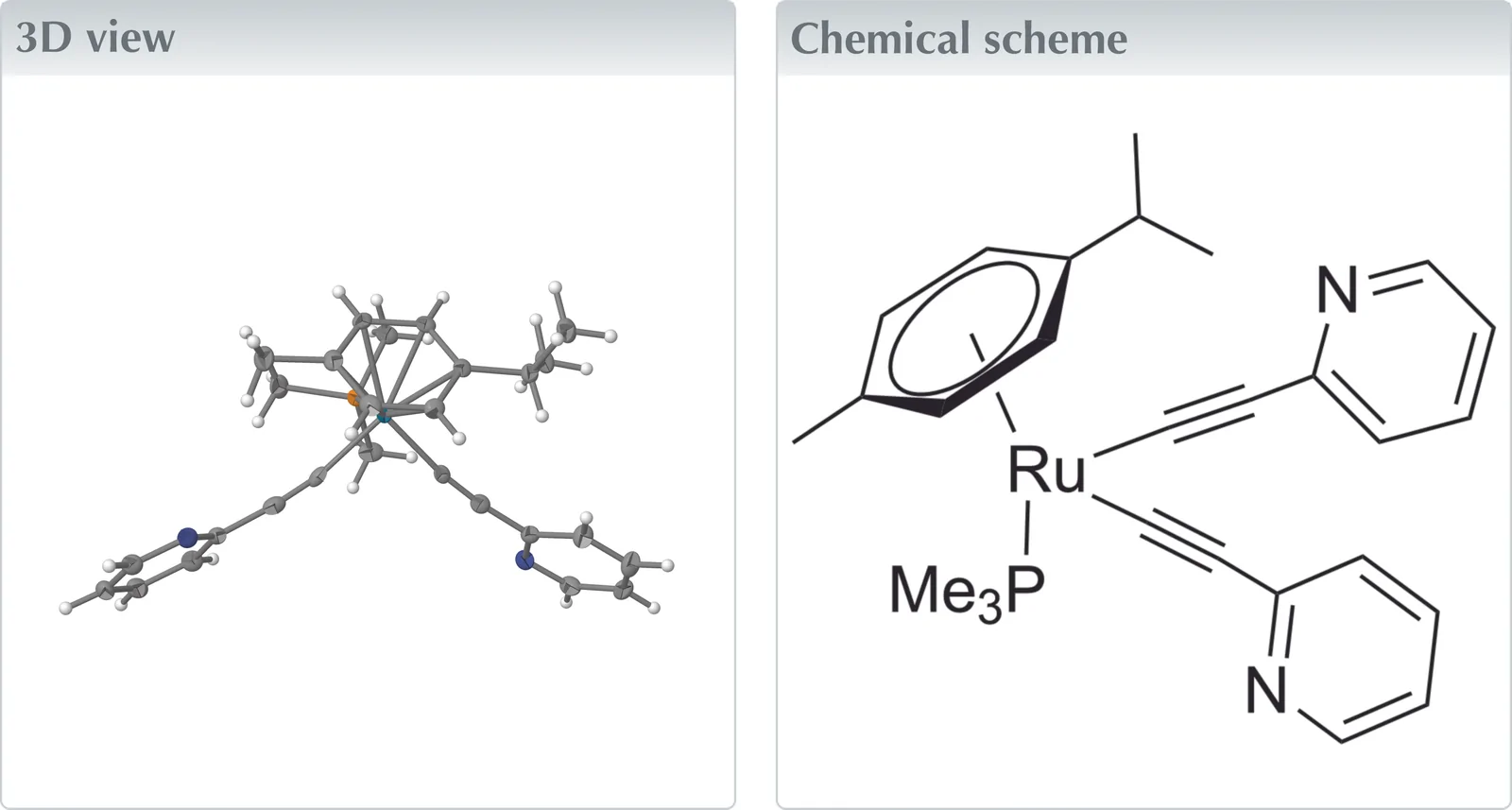

## Structure description

The title compound (**I**), where *p*-cym = η^6^-1-isopropyl-4-methyl­benzene and C_2_-2py = η^1^-pyridin-2-ylethynide, represents a newly characterized bis­alkynyl species. Alkynyl compounds have a variety of applications (Long & Williams, 2003 ▸), and our group has been investigating these species as ligands. In a previously reported titanocene, (Cp*)_2_Ti(C_2_-2py)_2_ where Cp* = penta­methyl­cyclo­penta­dienyl, it was found that the C—Ti—C bond angle was 90.3 (1)° and this metalloligand can coordinate Pd^II^ in a *trans*-bidentate fashion (Vieira *et al.*, 2017 ▸). Our initial hypothesis was that other transition-metal compounds with similarly oriented alkynyl­pyridine ligands could act as *trans*-bidentate ligands. We therefore focused on *cis*-alkynyl­pyridine ligands on square-planar and octa­hedral metal centers. For instance, we structurally characterized a polymorph of (^tbu^bpy)Pt(C_2_-2py)_2_ (where ^tbu^bpy = 4,4′-di-*tert*-butyl-2,2′-bi­pyridine) that has six unique mol­ecules in the asymmetric unit, with the C—Pt—C bond angles ranging from 88.8 (8) to 91.7 (8)° (Jaques *et al.*, 2020 ▸). Although the C—Pt—C angles are like the C—Ti—C angle in the (Cp*)_2_Ti(C_2_-2py)_2_ metalloligand, the Pt^II^ alkynyl­pyridine compound did not act as a *trans*-bidentate ligand under the conditions tested. Efforts are ongoing to investigate the coordination chemistry of (*p*-cym)Ru(C_2_-2py)_2_(PMe_3_) (**I**), and herein we describe structural details of this mol­ecule.

**Figure 1:**
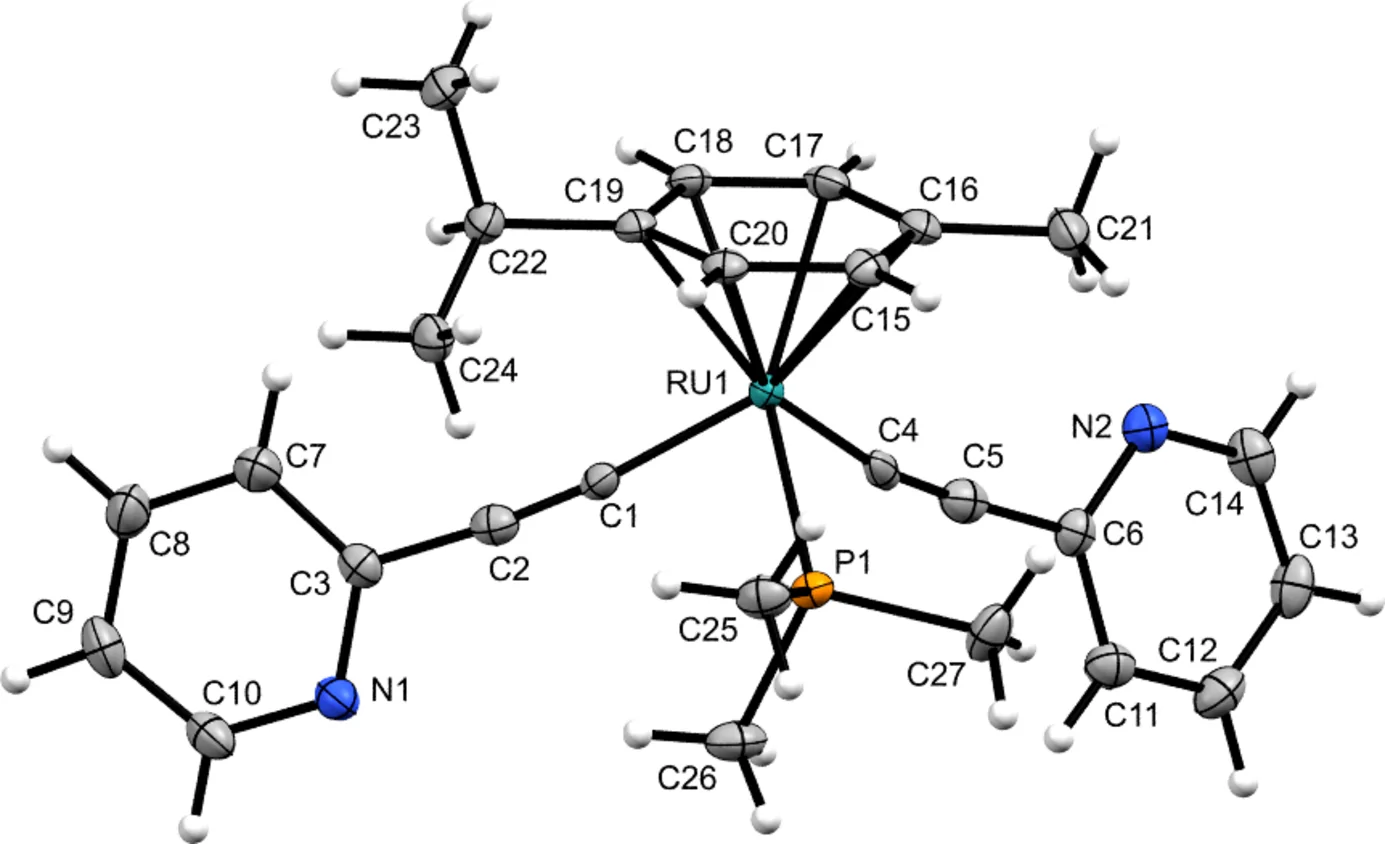
Displacement ellipsoid perspective view (50% probability) for the title structure showing the atom-numbering scheme.

The crystal structure of (*p*-cym)Ru(C_2_-2py)_2_(PMe_3_) (**I**) contains a pseudo-octa­hedral Ru^II^ atom with *p*-cym occupying one face of the octa­hedron and another face comprised of monodentate PMe_3_ and two η^1^-C_2_-2py ligands (Fig. 1 ▸). The angles about the Ru center involving the monodentate ligands are: 91.01 (6)° (C1—Ru1—C4), 87.22 (4)° (C1—Ru—P1), and 83.49 (4)° (C4—Ru1—P)1, which highlight the pseudo-octa­hedral half-sandwich or ‘piano-stool’ coordination of (**I**) (Lee *et al.*, 2016 ▸ and Davis and Muller, 2021 ▸). The Ru—C_ethynide_ bond lengths for (**I**) are 2.0248 (16) Å and 2.0343 (17) Å for Ru1—C1 and Ru1—C4, respectively. The Ru—C_ethnyide_ bond lengths along with the C≡C bond lengths, 1.192 (2) Å and 1.167 (3) Å, are in good agreement with a single resonance structure for (**I**), showing no evidence of Ru—C multiple-bond character. The similarly structured (*p*-cym)Ru(C_2_Ph)_2_(PMe_3_) (where C_2_Ph = η^1^-phenyl­ethynide) has Ru—C bond lengths of 2.003 (9) and 2.027 (7) Å, and a C—Ru—C bond angle of 91.5 (3)° (Menéndez *et al.*, 2001 ▸). In (**I**), the two pyridine rings appear to be in an anti-conformation upon visual inspection of the nitro­gen atoms, and this is qu­anti­fied by measuring the through space torsion angle between N1—C3—C6—N2 to be 151.1 (2)°. An analogous *anti*-conformation also occurs in the pyridyl groups in (Cp*)_2_Ti(C_2_-2py)_2_ (Vieira *et al.*, 2017 ▸). The Ru—C_*p*-cym_ bond lengths show little variation ranging from 2.2436 (15) Å to 2.2698 (16) Å and averaging 2.2601 (16) Å. The aromatic C—C bond distances in the facially coordinating *p*-cym ligand range from 1.400 (2) to 1.436 (2) Å, which includes three short and three long lengths that alternate. The three short lengths are approximately *trans* to the three monodentate ligands, which suggests some *trans*-effect from the PMe_3_ and C_*2*_-py ligands.

**Figure 2:**
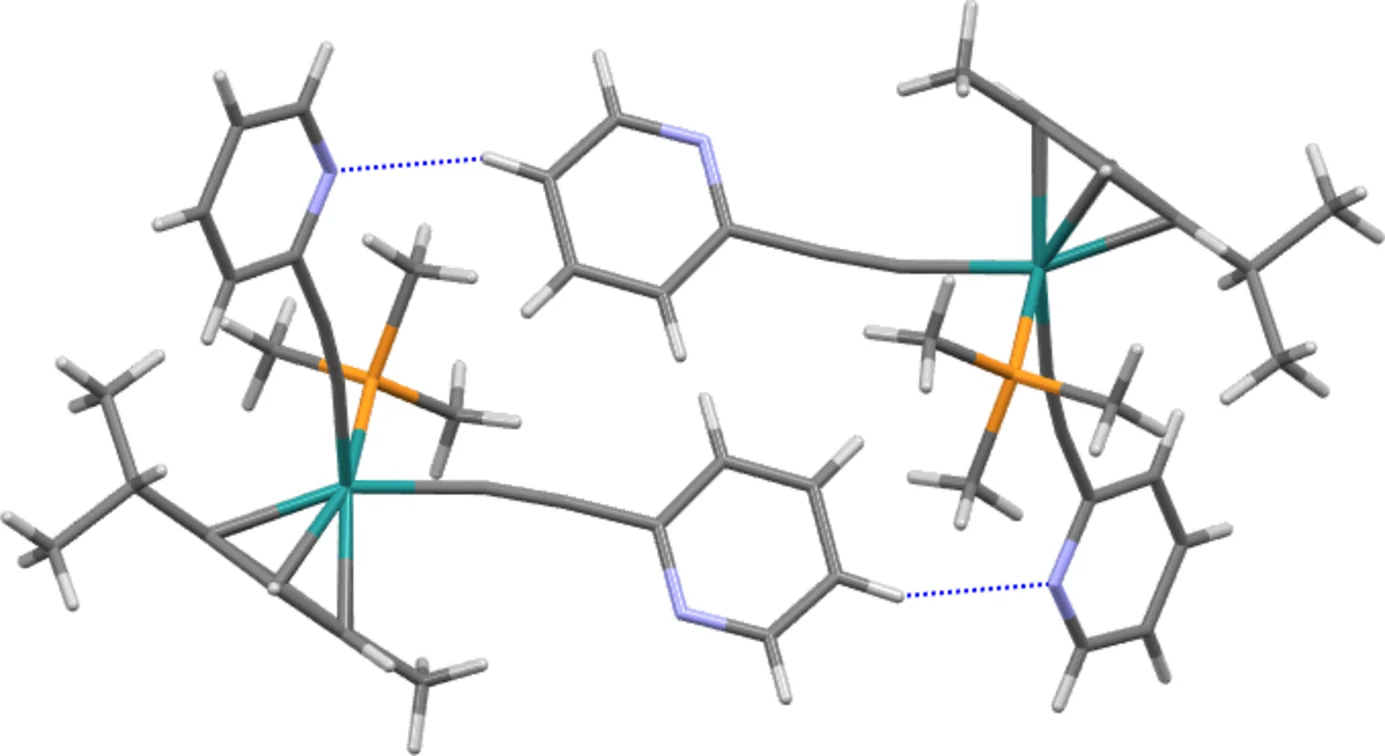
Inter­molecular hydrogen bonding forming a chain propagating along the *a*-axis direction where hydrogen bonds are represented by dashed lines.

**Figure 3:**
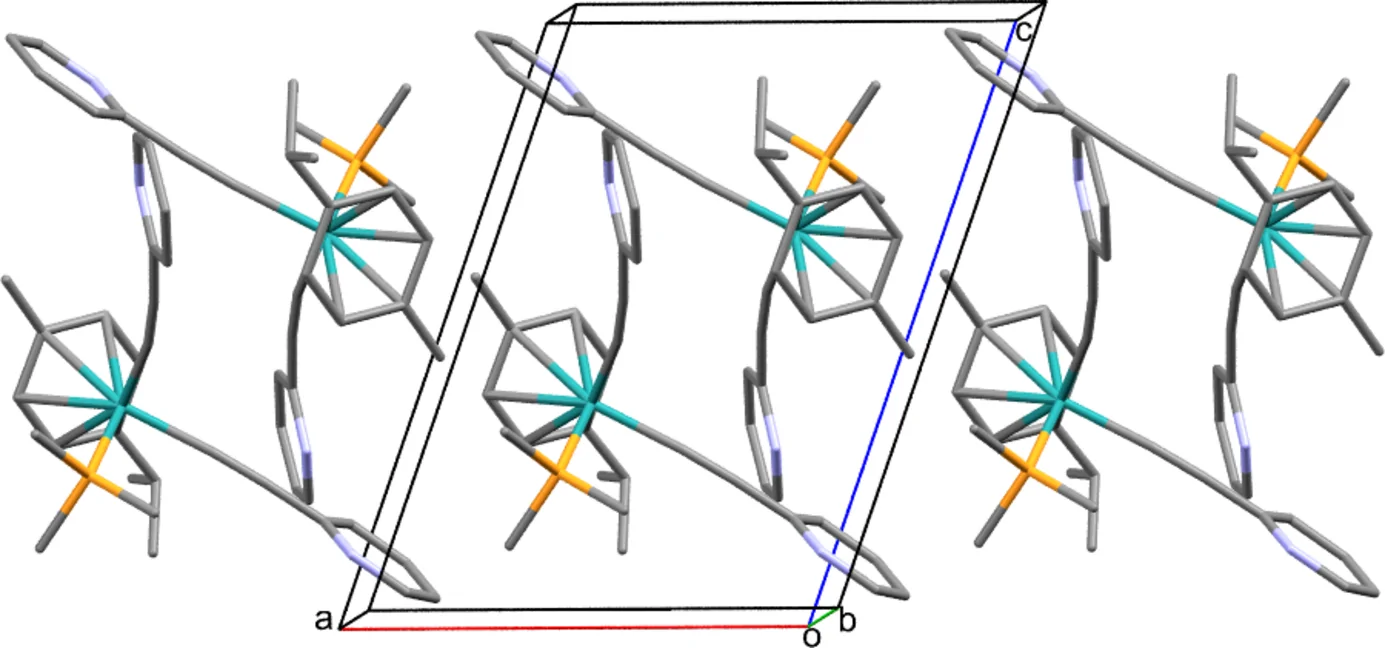
Extended structure viewed slightly off the *b* axis.

In the unit cell of (**I**), *Z* = 2 and those complexes are related by a center of inversion and are linked by two C_py_—H_*para*_⋯N_py_ hydrogen bonds with an average bond angle of 155.9 (2)° (Table 1 ▸), which suggests evidence of inter­mediate hydrogen bonding (Arunan *et al.*, 2011 ▸). The hydrogen bonding involves one C—H donor and one nitro­gen acceptor from each pyridine of the compound, which generates dimers of the complexes (Fig. 2 ▸). These inter­actions may influence a slight bending of the alkynyl­pyridine ligands, with Ru1—C1≡C2 of 172.55 (14)°, C1≡C2—C3 of 173.52 (17)°, Ru1—C4≡C5 of 168.85 (14)°, and C4≡C5—C6 of 174.28 (17)°. Translationally equivalent, adjacent dimers propagate in a chain along the *a*-axis direction (Fig. 3 ▸), primarily through C—H⋯π inter­actions (Table 1 ▸).

## Synthesis and crystallization

An oven-dried 50-ml Schlenk flask was brought into the glovebox and charged with a 1.00 *M* solution of 2-ethynyl­pyridine in tetra­hydro­furan (2.07 ml). After being sealed, the flask was removed from the glovebox and placed on a N_2_ Schlenk line and diluted with tetra­hydro­furan (10 ml) *via* syringe. After being cooled to −94 °C, the solution was treated with *n*-BuLi (0.970 ml, 2.09 mmol). The dark brown solution was stirred at −94 °C for 10 min and then brought to room temperature. To this solution, (*p*-cym)Ru(PMe_3_)(Cl)_2_ (0.2010 g, 0.5258 mmol) was added, and the reddish-brown homogenous mixture was allowed to stir at room temperature for 4 h. The solvent was removed on the rotary evaporator leaving a dark brown oil, and the residue was dissolved in di­chloro­methane and filtered through celite. The volatiles were reduced to dryness leaving a dark brown residue which was then triturated using diethyl ether. The final dark brown powder was collected on a Hirsch funnel dried and *in vacuo*. (0.254 g, 93.8% yield). ^1^H-NMR (400 MHz, chloro­form-*d*) δ 8.38 (*d*, *J* = 4.4 Hz, 2H), 7.41 (*td*, *J* = 7.7, 1.5 Hz, 2H), 7.22 (*d*, *J* = 7.9 Hz, 2H), 6.90 (overlapping *ddd*, *J* = 7.3, 4.9, and 1.3 Hz 2H), 5.64 (*d*, *J* = 6.1 Hz, 2H), 5.58 (*d*, *J* = 6.0 Hz, 2H), 2.89–2.86 (*sept*, *J* = 6.8 Hz, 1H), 2.29 (*s*, 3H), 1.69 (*d*, ^2^*J*_HP_ = 10.7 Hz, 9H), 1.29 (*d*, *J* = 6.9 Hz, 6H). ^13^C-NMR (101 MHz, chloro­form-*d*) δ 149.0, 147.2, 135.2, 128.9, 126.7, 118.9, 115.9, 104.3, 94.1 (*d*, ^3^*J_CP_* = 3.9 Hz), 91.7 (*d*, ^3^*J*_CP_ = 4.3 Hz), 31.7, 22.9, 19.6 (*d*, ^1^*J*_CP_ = 36.6 Hz), 19.3. ^31^P NMR (161 MHz, chloro­form-*d*) δ −15.3 (*s*). Analysis calculated (found) for (*p*-cym)Ru(PMe_3_)(C_2_-2-pyridine)_2_·H_2_O: C, 60.27 (60.40); H, 6.19 (5.87); N, 5.25 (5.74). IR ν_C≡C_ 2086 cm^−1^.

Crystals were obtained by layering a di­chloro­methane solution of (**I**) with hexa­nes in the dark, yielding yellow plates.

## Refinement

Crystal data, data collection, and structure refinement details are summarized in Table 2 ▸.

## Supplementary Material

Crystal structure: contains datablock(s) I. DOI: 10.1107/S241431462601045X/bt4209sup1.cif

Structure factors: contains datablock(s) I. DOI: 10.1107/S241431462601045X/bt4209Isup2.hkl

CCDC reference: 2594178

Additional supporting information:  crystallographic information; 3D view; checkCIF report

## Figures and Tables

**Table 1:** Hydrogen-bond geometry (Å, °) *Cg*1 and *Cg*2 are the centroids of the C3/C7–C10/N1 and C6/C11–C14/N2 rings, respectively.

*D*—H⋯*A*	*D*—H	H⋯*A*	*D*⋯*A*	*D*—H⋯*A*
C7—H7⋯N2^i^	0.95	2.64	3.556 (2)	161
C13—H13⋯N1^ii^	0.95	2.57	3.430 (2)	151
C20—H20⋯*Cg*1^iii^	0.95	3.17	3.94	139
C27—H27*B*⋯*Cg*2^iv^	0.98	2.85	3.70	145

Symmetry codes: (i) 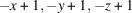; (ii) 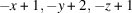; (iii) 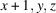; (iv) 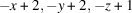.

**Table 2:** Experimental details

Crystal data	
Chemical formula	[Ru(C_7_H_4_N_2_)_2_(C_10_H_14_)(C_3_H_9_P)]
*M*_r_	515.58
Crystal system, space group	Triclinic, *P* 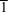
Temperature (K)	100
*a*, *b*, *c* (Å)	9.6665 (4), 10.7200 (5), 13.1717 (6)
α, β, γ (°)	101.9906 (12), 103.8890 (12), 108.3744 (12)
*V* (Å^3^)	1196.47 (9)
*Z*	2
Radiation type	Cu *K*α
μ (mm^−1^)	6.05
Crystal size (mm)	0.22 × 0.19 × 0.05

Data collection	
Diffractometer	Bruker D8 Venture Photon 2
Absorption correction	Multi-scan (*SADABS*; Krause *et al.*, 2015 ▸)
*T*_min_, *T*_max_	0.624, 1.000
No. of measured, independent and observed [*I* > 2σ(*I*)] reflections	38204, 4859, 4770
*R*_int_	0.040
(sin θ/λ)_max_ (Å^−1^)	0.625

Refinement	
*R*[*F*^2^ > 2σ(*F*^2^)], *wR*(*F*^2^), *S*	0.020, 0.051, 1.07
No. of reflections	4859
No. of parameters	286
H-atom treatment	H-atom parameters constrained
Δρ_max_, Δρ_min_ (e Å^−3^)	0.52, −0.31

Computer programs: *APEX3* and *SAINT* (Bruker, 2017 ▸), *SHELXT2014/5* (Sheldrick, 2015*a* ▸), *SHELXL2016/6* (Sheldrick, 2015*b* ▸), *Mercury* (Macrae *et al.*, 2020 ▸) and *publCIF* (Westrip, 2010 ▸).

## full crystallographic data

### (η6-1-Isopropyl-4-methylbenzene)bis(η1-pyridin-2-ylethynido-κC)(trimethylphosphane-κP)ruthenium(II) Crystal data

**Table:**

[Ru(C_7_H_4_N_2_)_2_(C_10_H_14_)(C_3_H_9_P)]	*Z* = 2
*M**_r_* = 515.58	*F*(000) = 532
Triclinic, *P*1	*D*_x_ = 1.431 Mg m^−^^3^
*a* = 9.6665 (4) Å	Cu *K*α radiation, λ = 1.54178 Å
*b* = 10.7200 (5) Å	Cell parameters from 9272 reflections
*c* = 13.1717 (6) Å	θ = 5.2–74.6°
α = 101.9906 (12)°	µ = 6.05 mm^−^^1^
β = 103.8890 (12)°	*T* = 100 K
γ = 108.3744 (12)°	Plate, yellow
*V* = 1196.47 (9) Å^3^	0.22 × 0.19 × 0.05 mm

### (η6-1-Isopropyl-4-methylbenzene)bis(η1-pyridin-2-ylethynido-κC)(trimethylphosphane-κP)ruthenium(II) Data collection

**Table:**

Bruker D8 Venture Photon 2 diffractometer	4770 reflections with *I* > 2σ(*I*)
Radiation source: Incoatec IµS	*R*_int_ = 0.040
φ and ω scans	θ_max_ = 74.7°, θ_min_ = 4.9°
Absorption correction: multi-scan (SADABS; Krause *et al.*, 2015)	*h* = −12→12
*T*_min_ = 0.624, *T*_max_ = 1.000	*k* = −13→13
38204 measured reflections	*l* = −16→16
4859 independent reflections	

### (η6-1-Isopropyl-4-methylbenzene)bis(η1-pyridin-2-ylethynido-κC)(trimethylphosphane-κP)ruthenium(II) Refinement

**Table:**

Refinement on *F*^2^	Primary atom site location: dual
Least-squares matrix: full	Secondary atom site location: difference Fourier map
*R*[*F*^2^ > 2σ(*F*^2^)] = 0.020	Hydrogen site location: inferred from neighbouring sites
*wR*(*F*^2^) = 0.051	H-atom parameters constrained
*S* = 1.07	*w* = 1/[σ^2^(*F*_o_^2^) + (0.021*P*)^2^ + 0.8929*P*] where *P* = (*F*_o_^2^ + 2*F*_c_^2^)/3
4859 reflections	(Δ/σ)_max_ = 0.002
286 parameters	Δρ_max_ = 0.52 e Å^−^^3^
0 restraints	Δρ_min_ = −0.31 e Å^−^^3^

### (η6-1-Isopropyl-4-methylbenzene)bis(η1-pyridin-2-ylethynido-κC)(trimethylphosphane-κP)ruthenium(II) Special details

**Table:**

Geometry. All esds (except the esd in the dihedral angle between two l.s. planes) are estimated using the full covariance matrix. The cell esds are taken into account individually in the estimation of esds in distances, angles and torsion angles; correlations between esds in cell parameters are only used when they are defined by crystal symmetry. An approximate (isotropic) treatment of cell esds is used for estimating esds involving l.s. planes.
Refinement. Hydrogen atoms were located in a difference map and refined as riding on their parent atom with C_tertiary_—H=1.00 Å, C_methyl_—H=0.98 Å, C_aromatic_—H=0.95 Å and with *U*(*H*)=1.5*U*_eq_(C_methyl_) and with *U*(H)=1.2*U*_eq_(C) for the remaining H atoms. The methyl groups were allowed to rotate but not to tip.

### (η6-1-Isopropyl-4-methylbenzene)bis(η1-pyridin-2-ylethynido-κC)(trimethylphosphane-κP)ruthenium(II) Fractional atomic coordinates and isotropic or equivalent isotropic displacement parameters (Å^2^)

**Table:**

	*x*	*y*	*z*	*U*_iso_*/*U*_eq_	
Ru1	0.65563 (2)	0.57466 (2)	0.35391 (2)	0.01282 (5)	
P1	0.68105 (5)	0.70169 (4)	0.23566 (3)	0.01634 (9)	
N1	0.04764 (17)	0.42475 (15)	0.11000 (12)	0.0223 (3)	
N2	0.79917 (16)	0.99076 (15)	0.70535 (12)	0.0218 (3)	
C1	0.42457 (18)	0.50340 (15)	0.27888 (13)	0.0164 (3)	
C2	0.2896 (2)	0.44745 (17)	0.22983 (14)	0.0209 (3)	
C3	0.12987 (19)	0.36582 (17)	0.16778 (13)	0.0191 (3)	
C4	0.65350 (17)	0.74745 (17)	0.45178 (13)	0.0178 (3)	
C5	0.67684 (19)	0.85009 (18)	0.51729 (14)	0.0222 (3)	
C6	0.72193 (18)	0.97960 (17)	0.60112 (14)	0.0191 (3)	
C7	0.0635 (2)	0.22850 (18)	0.16713 (15)	0.0255 (4)	
H7	0.122521	0.189822	0.209549	0.031*	
C8	−0.0891 (2)	0.15055 (19)	0.10384 (16)	0.0295 (4)	
H8	−0.135719	0.057181	0.101568	0.035*	
C9	−0.1732 (2)	0.2098 (2)	0.04383 (15)	0.0277 (4)	
H9	−0.278201	0.158431	−0.000273	0.033*	
C10	−0.0996 (2)	0.34637 (19)	0.05004 (14)	0.0253 (4)	
H10	−0.157649	0.386918	0.008953	0.030*	
C11	0.6894 (2)	1.09009 (18)	0.57537 (15)	0.0241 (3)	
H11	0.634210	1.079933	0.502009	0.029*	
C12	0.7381 (2)	1.21395 (18)	0.65723 (16)	0.0272 (4)	
H12	0.715797	1.289192	0.640898	0.033*	
C13	0.8195 (2)	1.22657 (18)	0.76312 (16)	0.0269 (4)	
H13	0.856097	1.310705	0.820917	0.032*	
C14	0.8461 (2)	1.11201 (19)	0.78222 (15)	0.0253 (4)	
H14	0.901539	1.120657	0.855123	0.030*	
C15	0.88305 (18)	0.55396 (17)	0.36949 (14)	0.0188 (3)	
H15	0.965175	0.604791	0.348262	0.023*	
C16	0.88120 (19)	0.60241 (17)	0.47651 (14)	0.0201 (3)	
C17	0.75568 (19)	0.52310 (17)	0.50552 (13)	0.0201 (3)	
H17	0.751468	0.553678	0.577356	0.024*	
C18	0.63897 (19)	0.40128 (16)	0.43001 (13)	0.0182 (3)	
H18	0.558205	0.349378	0.451854	0.022*	
C19	0.63979 (18)	0.35378 (16)	0.31992 (13)	0.0163 (3)	
C20	0.76420 (18)	0.42991 (16)	0.29173 (13)	0.0175 (3)	
H20	0.769427	0.398682	0.220300	0.021*	
C21	1.0031 (2)	0.73548 (19)	0.55802 (15)	0.0275 (4)	
H21A	1.068749	0.714880	0.615974	0.041*	
H21B	0.953339	0.792175	0.590703	0.041*	
H21C	1.066314	0.785909	0.520383	0.041*	
C22	0.51048 (19)	0.22202 (16)	0.24052 (14)	0.0198 (3)	
H22	0.417857	0.209447	0.264708	0.024*	
C23	0.5567 (2)	0.09907 (17)	0.24827 (16)	0.0272 (4)	
H23A	0.646335	0.107754	0.223677	0.041*	
H23B	0.470309	0.012939	0.201463	0.041*	
H23C	0.583068	0.097968	0.324629	0.041*	
C24	0.4643 (2)	0.22366 (18)	0.12171 (14)	0.0261 (4)	
H24A	0.438572	0.304471	0.118535	0.039*	
H24B	0.374345	0.139320	0.076537	0.039*	
H24C	0.550501	0.228240	0.093759	0.039*	
C25	0.7288 (2)	0.62688 (18)	0.11774 (14)	0.0237 (3)	
H25A	0.646330	0.536955	0.074119	0.036*	
H25B	0.826224	0.614828	0.143528	0.036*	
H25C	0.739483	0.688679	0.072233	0.036*	
C26	0.5155 (2)	0.7375 (2)	0.17165 (16)	0.0275 (4)	
H26A	0.485104	0.785860	0.228718	0.041*	
H26B	0.429445	0.650470	0.126320	0.041*	
H26C	0.542677	0.795633	0.125343	0.041*	
C27	0.8338 (2)	0.87270 (18)	0.29789 (16)	0.0284 (4)	
H27A	0.844269	0.918281	0.241322	0.043*	
H27B	0.931295	0.864499	0.331484	0.043*	
H27C	0.808136	0.927576	0.354418	0.043*	

### (η6-1-Isopropyl-4-methylbenzene)bis(η1-pyridin-2-ylethynido-κC)(trimethylphosphane-κP)ruthenium(II) Atomic displacement parameters (Å^2^)

**Table:**

	*U*^11^	*U*^22^	*U*^33^	*U*^12^	*U*^13^	*U*^23^
Ru1	0.01285 (7)	0.01244 (7)	0.01278 (7)	0.00485 (5)	0.00363 (5)	0.00378 (4)
P1	0.01931 (19)	0.01539 (18)	0.01715 (18)	0.00801 (15)	0.00732 (15)	0.00705 (15)
N1	0.0222 (7)	0.0218 (7)	0.0207 (7)	0.0100 (6)	0.0030 (6)	0.0044 (5)
N2	0.0206 (7)	0.0223 (7)	0.0220 (7)	0.0077 (6)	0.0073 (6)	0.0064 (6)
C1	0.0215 (8)	0.0130 (7)	0.0158 (7)	0.0063 (6)	0.0075 (6)	0.0054 (6)
C2	0.0241 (9)	0.0201 (8)	0.0215 (8)	0.0109 (7)	0.0081 (7)	0.0082 (6)
C3	0.0189 (8)	0.0216 (8)	0.0168 (7)	0.0086 (6)	0.0055 (6)	0.0052 (6)
C4	0.0114 (7)	0.0229 (8)	0.0162 (7)	0.0015 (6)	0.0024 (6)	0.0107 (6)
C5	0.0183 (8)	0.0279 (9)	0.0260 (8)	0.0104 (7)	0.0105 (7)	0.0138 (7)
C6	0.0182 (7)	0.0192 (8)	0.0205 (8)	0.0061 (6)	0.0095 (6)	0.0053 (6)
C7	0.0244 (9)	0.0247 (9)	0.0245 (8)	0.0071 (7)	0.0044 (7)	0.0096 (7)
C8	0.0271 (9)	0.0244 (9)	0.0290 (9)	0.0013 (7)	0.0068 (8)	0.0084 (7)
C9	0.0172 (8)	0.0337 (10)	0.0224 (8)	0.0050 (7)	0.0026 (7)	0.0008 (7)
C10	0.0237 (8)	0.0310 (9)	0.0194 (8)	0.0148 (7)	0.0020 (7)	0.0032 (7)
C11	0.0258 (9)	0.0253 (8)	0.0238 (8)	0.0105 (7)	0.0097 (7)	0.0100 (7)
C12	0.0298 (9)	0.0208 (8)	0.0367 (10)	0.0118 (7)	0.0167 (8)	0.0104 (7)
C13	0.0253 (9)	0.0218 (8)	0.0291 (9)	0.0053 (7)	0.0136 (7)	−0.0010 (7)
C14	0.0228 (8)	0.0289 (9)	0.0208 (8)	0.0079 (7)	0.0082 (7)	0.0029 (7)
C15	0.0149 (7)	0.0195 (8)	0.0259 (8)	0.0092 (6)	0.0076 (6)	0.0096 (6)
C16	0.0176 (8)	0.0201 (8)	0.0208 (8)	0.0093 (6)	0.0004 (6)	0.0065 (6)
C17	0.0237 (8)	0.0242 (8)	0.0150 (7)	0.0129 (7)	0.0037 (6)	0.0083 (6)
C18	0.0205 (8)	0.0188 (7)	0.0201 (8)	0.0096 (6)	0.0070 (6)	0.0117 (6)
C19	0.0181 (7)	0.0144 (7)	0.0185 (7)	0.0088 (6)	0.0048 (6)	0.0067 (6)
C20	0.0196 (8)	0.0182 (7)	0.0197 (7)	0.0120 (6)	0.0074 (6)	0.0074 (6)
C21	0.0218 (8)	0.0255 (9)	0.0257 (9)	0.0075 (7)	−0.0021 (7)	0.0026 (7)
C22	0.0191 (8)	0.0158 (7)	0.0221 (8)	0.0059 (6)	0.0052 (6)	0.0042 (6)
C23	0.0284 (9)	0.0171 (8)	0.0325 (9)	0.0094 (7)	0.0047 (8)	0.0054 (7)
C24	0.0275 (9)	0.0226 (8)	0.0206 (8)	0.0093 (7)	0.0002 (7)	0.0007 (7)
C25	0.0309 (9)	0.0268 (9)	0.0201 (8)	0.0150 (7)	0.0125 (7)	0.0100 (7)
C26	0.0307 (9)	0.0316 (9)	0.0337 (10)	0.0199 (8)	0.0146 (8)	0.0202 (8)
C27	0.0339 (10)	0.0194 (8)	0.0293 (9)	0.0028 (7)	0.0156 (8)	0.0081 (7)

### (η6-1-Isopropyl-4-methylbenzene)bis(η1-pyridin-2-ylethynido-κC)(trimethylphosphane-κP)ruthenium(II) Geometric parameters (Å, º)

**Table:**

Ru1—C1	2.0248 (16)	C14—H14	0.9500
Ru1—C4	2.0343 (17)	C15—C16	1.404 (2)
Ru1—C15	2.2436 (15)	C15—C20	1.429 (2)
Ru1—C20	2.2563 (15)	C15—H15	0.9500
Ru1—C17	2.2577 (15)	C16—C17	1.431 (2)
Ru1—C19	2.2646 (15)	C16—C21	1.507 (2)
Ru1—C18	2.2688 (15)	C17—C18	1.400 (2)
Ru1—C16	2.2698 (16)	C17—H17	0.9500
Ru1—P1	2.2792 (4)	C18—C19	1.436 (2)
P1—C26	1.8146 (18)	C18—H18	0.9500
P1—C27	1.8167 (18)	C19—C20	1.404 (2)
P1—C25	1.8244 (17)	C19—C22	1.516 (2)
N1—C10	1.336 (2)	C20—H20	0.9500
N1—C3	1.355 (2)	C21—H21A	0.9800
N2—C14	1.335 (2)	C21—H21B	0.9800
N2—C6	1.356 (2)	C21—H21C	0.9800
C1—C2	1.192 (2)	C22—C24	1.526 (2)
C2—C3	1.436 (2)	C22—C23	1.534 (2)
C3—C7	1.406 (2)	C22—H22	1.0000
C4—C5	1.167 (3)	C23—H23A	0.9800
C5—C6	1.445 (2)	C23—H23B	0.9800
C6—C11	1.403 (2)	C23—H23C	0.9800
C7—C8	1.382 (3)	C24—H24A	0.9800
C7—H7	0.9500	C24—H24B	0.9800
C8—C9	1.384 (3)	C24—H24C	0.9800
C8—H8	0.9500	C25—H25A	0.9800
C9—C10	1.387 (3)	C25—H25B	0.9800
C9—H9	0.9500	C25—H25C	0.9800
C10—H10	0.9500	C26—H26A	0.9800
C11—C12	1.384 (3)	C26—H26B	0.9800
C11—H11	0.9500	C26—H26C	0.9800
C12—C13	1.382 (3)	C27—H27A	0.9800
C12—H12	0.9500	C27—H27B	0.9800
C13—C14	1.391 (3)	C27—H27C	0.9800
C13—H13	0.9500		
			
C1—Ru1—C4	91.01 (6)	C16—C15—C20	121.52 (15)
C1—Ru1—C15	150.44 (6)	C16—C15—Ru1	72.89 (9)
C4—Ru1—C15	118.47 (6)	C20—C15—Ru1	71.96 (9)
C1—Ru1—C20	113.58 (6)	C16—C15—H15	119.2
C4—Ru1—C20	155.38 (6)	C20—C15—H15	119.2
C15—Ru1—C20	37.04 (6)	Ru1—C15—H15	128.2
C1—Ru1—C17	121.14 (6)	C15—C16—C17	117.65 (15)
C4—Ru1—C17	89.35 (6)	C15—C16—C21	121.98 (16)
C15—Ru1—C17	65.22 (6)	C17—C16—C21	120.34 (16)
C20—Ru1—C17	77.19 (6)	C15—C16—Ru1	70.87 (9)
C1—Ru1—C19	89.31 (6)	C17—C16—Ru1	71.11 (9)
C4—Ru1—C19	151.13 (6)	C21—C16—Ru1	127.27 (11)
C15—Ru1—C19	66.19 (6)	C18—C17—C16	121.24 (15)
C20—Ru1—C19	36.19 (6)	C18—C17—Ru1	72.42 (9)
C17—Ru1—C19	66.12 (6)	C16—C17—Ru1	72.03 (9)
C1—Ru1—C18	93.52 (6)	C18—C17—H17	119.4
C4—Ru1—C18	114.29 (6)	C16—C17—H17	119.4
C15—Ru1—C18	77.00 (6)	Ru1—C17—H17	128.5
C20—Ru1—C18	65.13 (6)	C17—C18—C19	120.84 (15)
C17—Ru1—C18	36.03 (6)	C17—C18—Ru1	71.55 (9)
C19—Ru1—C18	36.94 (6)	C19—C18—Ru1	71.37 (8)
C1—Ru1—C16	157.91 (6)	C17—C18—H18	119.6
C4—Ru1—C16	90.64 (6)	C19—C18—H18	119.6
C15—Ru1—C16	36.24 (6)	Ru1—C18—H18	130.1
C20—Ru1—C16	66.22 (6)	C20—C19—C18	118.07 (14)
C17—Ru1—C16	36.85 (6)	C20—C19—C22	122.77 (14)
C19—Ru1—C16	78.82 (6)	C18—C19—C22	119.12 (14)
C18—Ru1—C16	65.85 (6)	C20—C19—Ru1	71.58 (9)
C1—Ru1—P1	87.22 (4)	C18—C19—Ru1	71.69 (9)
C4—Ru1—P1	83.49 (4)	C22—C19—Ru1	129.53 (10)
C15—Ru1—P1	93.68 (4)	C19—C20—C15	120.63 (15)
C20—Ru1—P1	98.27 (4)	C19—C20—Ru1	72.23 (9)
C17—Ru1—P1	150.93 (5)	C15—C20—Ru1	71.00 (9)
C19—Ru1—P1	125.35 (4)	C19—C20—H20	119.7
C18—Ru1—P1	162.17 (4)	C15—C20—H20	119.7
C16—Ru1—P1	114.85 (4)	Ru1—C20—H20	129.6
C26—P1—C27	102.86 (9)	C16—C21—H21A	109.5
C26—P1—C25	102.51 (9)	C16—C21—H21B	109.5
C27—P1—C25	103.39 (9)	H21A—C21—H21B	109.5
C26—P1—Ru1	117.58 (6)	C16—C21—H21C	109.5
C27—P1—Ru1	113.81 (6)	H21A—C21—H21C	109.5
C25—P1—Ru1	114.87 (6)	H21B—C21—H21C	109.5
C10—N1—C3	117.44 (15)	C19—C22—C24	114.00 (14)
C14—N2—C6	117.44 (15)	C19—C22—C23	109.14 (13)
C2—C1—Ru1	172.55 (14)	C24—C22—C23	110.50 (14)
C1—C2—C3	173.52 (17)	C19—C22—H22	107.7
N1—C3—C7	121.84 (15)	C24—C22—H22	107.7
N1—C3—C2	118.06 (15)	C23—C22—H22	107.7
C7—C3—C2	120.10 (15)	C22—C23—H23A	109.5
C5—C4—Ru1	168.85 (14)	C22—C23—H23B	109.5
C4—C5—C6	174.28 (17)	H23A—C23—H23B	109.5
N2—C6—C11	121.38 (15)	C22—C23—H23C	109.5
N2—C6—C5	117.32 (15)	H23A—C23—H23C	109.5
C11—C6—C5	121.30 (15)	H23B—C23—H23C	109.5
C8—C7—C3	119.03 (16)	C22—C24—H24A	109.5
C8—C7—H7	120.5	C22—C24—H24B	109.5
C3—C7—H7	120.5	H24A—C24—H24B	109.5
C7—C8—C9	119.38 (17)	C22—C24—H24C	109.5
C7—C8—H8	120.3	H24A—C24—H24C	109.5
C9—C8—H8	120.3	H24B—C24—H24C	109.5
C8—C9—C10	117.98 (16)	P1—C25—H25A	109.5
C8—C9—H9	121.0	P1—C25—H25B	109.5
C10—C9—H9	121.0	H25A—C25—H25B	109.5
N1—C10—C9	124.33 (16)	P1—C25—H25C	109.5
N1—C10—H10	117.8	H25A—C25—H25C	109.5
C9—C10—H10	117.8	H25B—C25—H25C	109.5
C12—C11—C6	119.75 (16)	P1—C26—H26A	109.5
C12—C11—H11	120.1	P1—C26—H26B	109.5
C6—C11—H11	120.1	H26A—C26—H26B	109.5
C13—C12—C11	119.02 (17)	P1—C26—H26C	109.5
C13—C12—H12	120.5	H26A—C26—H26C	109.5
C11—C12—H12	120.5	H26B—C26—H26C	109.5
C12—C13—C14	117.79 (16)	P1—C27—H27A	109.5
C12—C13—H13	121.1	P1—C27—H27B	109.5
C14—C13—H13	121.1	H27A—C27—H27B	109.5
N2—C14—C13	124.60 (17)	P1—C27—H27C	109.5
N2—C14—H14	117.7	H27A—C27—H27C	109.5
C13—C14—H14	117.7	H27B—C27—H27C	109.5
			
C10—N1—C3—C7	−1.2 (2)	C15—C16—C17—Ru1	−55.22 (13)
C10—N1—C3—C2	178.07 (15)	C21—C16—C17—Ru1	122.78 (15)
C14—N2—C6—C11	−1.4 (2)	C16—C17—C18—C19	−1.5 (2)
C14—N2—C6—C5	177.52 (15)	Ru1—C17—C18—C19	53.73 (13)
N1—C3—C7—C8	1.4 (3)	C16—C17—C18—Ru1	−55.21 (14)
C2—C3—C7—C8	−177.84 (17)	C17—C18—C19—C20	2.6 (2)
C3—C7—C8—C9	−0.8 (3)	Ru1—C18—C19—C20	56.38 (12)
C7—C8—C9—C10	0.0 (3)	C17—C18—C19—C22	−179.48 (14)
C3—N1—C10—C9	0.4 (3)	Ru1—C18—C19—C22	−125.66 (13)
C8—C9—C10—N1	0.2 (3)	C17—C18—C19—Ru1	−53.82 (13)
N2—C6—C11—C12	0.7 (3)	C18—C19—C20—C15	−2.4 (2)
C5—C6—C11—C12	−178.22 (16)	C22—C19—C20—C15	179.73 (14)
C6—C11—C12—C13	0.6 (3)	Ru1—C19—C20—C15	54.04 (13)
C11—C12—C13—C14	−1.1 (3)	C18—C19—C20—Ru1	−56.44 (12)
C6—N2—C14—C13	0.9 (3)	C22—C19—C20—Ru1	125.69 (14)
C12—C13—C14—N2	0.4 (3)	C16—C15—C20—C19	1.2 (2)
C20—C15—C16—C17	0.0 (2)	Ru1—C15—C20—C19	−54.61 (13)
Ru1—C15—C16—C17	55.35 (13)	C16—C15—C20—Ru1	55.77 (13)
C20—C15—C16—C21	−177.96 (15)	C20—C19—C22—C24	−36.8 (2)
Ru1—C15—C16—C21	−122.62 (15)	C18—C19—C22—C24	145.31 (15)
C20—C15—C16—Ru1	−55.34 (13)	Ru1—C19—C22—C24	55.7 (2)
C15—C16—C17—C18	0.2 (2)	C20—C19—C22—C23	87.25 (18)
C21—C16—C17—C18	178.17 (15)	C18—C19—C22—C23	−90.61 (18)
Ru1—C16—C17—C18	55.39 (14)	Ru1—C19—C22—C23	179.77 (12)

### (η6-1-Isopropyl-4-methylbenzene)bis(η1-pyridin-2-ylethynido-κC)(trimethylphosphane-κP)ruthenium(II) Hydrogen-bond geometry (Å, º)

*Cg*1 and *Cg*2 are the centroids of the C3/C7–C10/N1 and C6/C11–C14/N2 rings, respectively.

**Table:**

*D*—H···*A*	*D*—H	H···*A*	*D*···*A*	*D*—H···*A*
C7—H7···N2^i^	0.95	2.64	3.556 (2)	161
C13—H13···N1^ii^	0.95	2.57	3.430 (2)	151
C20—H20···Cg1^iii^	0.95	3.17	3.94	139
C27—H27*B*···Cg2^iv^	0.98	2.85	3.70	145

Symmetry codes: (i) −*x*+1, −*y*+1, −*z*+1; (ii) −*x*+1, −*y*+2, −*z*+1; (iii) *x*+1, *y*, *z*; (iv) −*x*+2, −*y*+2, −*z*+1.
